# Physicochemical Characterization and Immunomodulatory Activity of a Novel Acid Polysaccharide from *Solanum muricatum*

**DOI:** 10.3390/polym11121972

**Published:** 2019-11-30

**Authors:** Heng Yue, Qianqian Xu, Xianheng Li, Jeevithan Elango, Wenhui Wu, Jianfeng Xu

**Affiliations:** 1 Department of Biopharmaceutics, College of Food Science and Technology, Shanghai Ocean University, Shanghai 201306, China; YHSHOU1234@163.COM (H.Y.); srijeevithan@gmail.com (J.E.); 2 Quality Supervision, Inspection and Testing Center for Cold Storage and Refrigeration Equipment, Ministry of Agriculture, Shanghai 201306, China

**Keywords:** polysaccharide, *Solanum muricatum*, purification, structural characterization, immunomodulatory activity

## Abstract

To investigate the structure and immunomodulatory activity of polysaccharide from *Solanum muricatum*, a novel acid polysaccharide named SMP-3a was purified from *Solanum muricatum* pulp through DEAE-52 cellulose column and Sephadex G-200 chromatography. Monosaccharide composition analysis showed that SMP-3a was mainly composed of rhamnose, arabinose, galactose, and galacturonic acid with the molar ratio of 1.09:2.64:1.54:1. The average molecular weight was found to be 227 kDa by high performance gel permeation chromatography (HPGPC). Thermal studies revealed the SMP-3a was a thermally stable polymer. Based on the results of methylation and NMR analysis, the backbone chain of SMP-3a was composed of →2)-α-l-Rha*p*-(1→, →4)-α-d-Gal*p*A-(1→ and →4)-α-d-Gal*p*-(1→. The side chain was consisted of α-l-Ara*f*-(1→ and →5)-α-l-Ara*f*-(1→. Immunomodulatory assay indicated that SMP-3a could significantly promote the proliferation of macrophages and stimulate the secretion of cytokines, including TNF-α, IL-1β, and IL-6. Our results suggested that SMP-3a could be used as a novel potential immunomodulatory agent in functional food.

## 1. Introduction

*Solanum muricatum*, which belongs to the Solanaceae family, is an herbaceous plant originally cultivated in the Andean region of South America and has been disseminated throughout the world. It is commonly called pepino or melon pear and can be grown under diverse soil and climatic conditions [1,2]. The color of *Solanum muricatum* begins green and changes to golden yellow covered with purple stripes during the ripening period [3]. This fruit is rich in minerals such as calcium, potassium, and phosphorous. It also contains vitamins such as ascorbic acid, niacin, riboflavin, and thiamin, which are ideal for a number of metabolic and antioxidant reactions [4]. The analysis of volatile aromatic constituents of *Solanum muricatum* have shown that *Solanum muricatum* contains terpenes and β-damascenone, which contributes to the exotic aromas of the food [5]. Besides, this plant food is rich in phenolic acids and flavonoids [6].

Polysaccharides are one kind of biological macromolecules composed of more than 10 monosaccharides. They are usually important and main bioactive constituents with complex structure of plants, fungi, and seaweed. In recent years, natural polysaccharides have attracted widespread attention due to their structural diversity, low toxicity, and unique biological activity, especially the immunomodulatory activity [7,8,9,10]. A large proportion of the current research focused on the activity of polysaccharides has shown that the natural polysaccharides extracted from plants possess a wide variety of biological activity, such as immunomodulatory, antitumor, and antioxidant effects [8,11,12]. The biological activity of polysaccharides is related to monosaccharide composition, molecular weight, glycosidic linkage, and chemical structure [13]. Thus, the systematic characterization of the structure of polysaccharides is an important first step for the study of biological activity on polysaccharides.

Previous studies on *Solanum muricatum* mainly involved the activity of aqueous and ethanol extracts [4,6]. However, studies on the polysaccharides from *Solanum muricatum* are limited. Therefore, this study aimed to obtain a purified polysaccharide from the pulp of *Solanum muricatum* using hot water extraction, to characterize the purified polysaccharide, and to study the immunoregulatory activity of the purified polysaccharide.

## 2. Materials and Methods

### 2.1. Materials and Reagents

The *Solanum muricatum* was purchased from a local supermarket in Wuwei City, Gansu Province of China. The *Solanum muricatum* was washed in flowing tap water to remove impurities. The washed *Solanum muricatum* was cut into thin slices and then dried in an electro-thermostatic blast oven (DHG-9053A, Shanghai Yiheng Scientific Instrument Co., Shanghai, China) at 50 °C. The dried *Solanum muricatum* was then crushed and sieved through a 60 mesh sieve to obtain *Solanum muricatum* powder.

DEAE-52 cellulose and Sephadex G-200 were purchased from Shanghai Yuanye Biotechnology Co. (Shanghai, China). Standard monosaccharides (rhamnose, fucose, arabinose, xylose, mannose, glucose, galactose) were purchased from Sigma Chemical Co. (St. Louis, MO, USA). Lipopolysaccharide (LPS) was obtained from Beyotime Institute of Biotechnology (Shanghai, China). Dulbecco’s modified Eagle medium (DMEM) and fetal bovine serum (FBS) were purchased from Thermofisher Scientific Inc. (Millersburg, PA, USA). CCK-8 was purchased from Dalian Meilun Biotech Co. (Dalian, China). ELISA kits for tumor necrosis factor (TNF-α), interleukin-1β (IL-1β), and interleukin-6 (IL-6) were all purchased from Nanjing Jiancheng Bioengineering Institute (Nanjing, China). All other reagents used were of analytical grade unless otherwise specified.

### 2.2. Extraction of Crude Polysaccharide (SMP)

The *Solanum muricatum* powder was treated with 95% ethanol at 80 °C for 6 h to remove lipids, pigments, monosaccharides, and small molecules. The residue was dried in an electro-thermostatic blast oven at 50 °C to obtain defatted *Solanum muricatum* powder. Ten grams of defatted powder was extracted three times with hot distilled water at 90 °C for 3.5 h. The extract solution was filtered in a Büchner funnel, concentrated to a suitable volume using a vacuum rotary evaporator and stored at 4 °C overnight. Then the filtrate was centrifuged at 10,000 rpm for 10 min to remove the residue. The supernatant was collected and mixed with four times volume of absolute ethanol for 10 h. After centrifugation at 12,000 rpm for 10 min, the crude polysaccharide SMP was obtained with the yield of 10.12% by lyophilized at −84 °C (Labconco Corporation, Kansas City, MO, USA).

### 2.3. Purification of SMP

After 1 g of the SMP was dissolved in 25 mL of the water, the solution was centrifuged at 10,000 rpm for 10 min to remove the residue. Then 25 mL 8% trichloroacetic acid (TCA) was added into the supernate to remove protein from SMP. The solution was poured in two dialysis bags (3500 MW) in a 2 L beaker for four days using magnetic stirrer, and the ultrapure water was changed every 6 h. After dialysis, the solution was lyophilized at −84 °C. The protein-removed SMP was purified sequentially by DEAE-52 anion-exchange chromatography and Sephadex G-200 gel filtration chromatography according to the reported method with little modifications [14].

Firstly, the SMP was completely dissolved in the ultrapure water. The solution was filtered (0.45 μm) and applied to a DEAE-52 cellulose anion-exchange column (3 × 45 cm). Then, the column was eluted with a stepwise NaCl gradient by 0, 0.05, 0.1, 0.3, 0.5, and 1 M NaCl solutions at a flow rate of 1mL/min. The eluate (10 mL/tube) was collected and detected by using the phenol-sulfuric acid method to determine polysaccharide [15]. The main fraction, SMP-3 was collected, dialyzed, and applied to a Sephadex G-200 gel filtration chromatography column (1.6 × 50 cm). The column was eluted with ultrapure water at a flow rate of 0.2 mL/min and the eluate (4 mL/tube) was collected and detected as described above. One purified fraction (SMP-3a) was finally collected for further study.

### 2.4. FT-IR Spectrum Analysis

FT-IR spectrum analysis of SMP-3a was performed at room temperature with a Fourier transform infrared spectrometer (Thermo Fisher Scientific, Waltham, MA, USA) in the range of 4000–400 cm^−1^. The dried SMP-3a (2 mg) was mixed with KBr powder and then pressed into 1 mm pellet for FT-IR analysis.

### 2.5. Determination of Molecular Weight

The molecular weight and homogeneity of SMP-3a were determined by high performance gel permeation chromatography (HPGPC) using Shimadzu HPLC system equipped with a series-connected KS805-804-802 column (7.8 mm × 300 mm) and a Shimadzu LC-10A RID detector. The columns were calibrated using standard dextrans with various molecular weights (5.2, 11.6, 148, 273, and 410 kDa) and eluted with 0.05 M NaCl at a flow rate of 0.8 mL/min. After calculating the logarithm of molecular weight on standard dextrans, a linear regression analysis was performed between the Lg (Mw) and retention time (Rt). The molecular weight of SMP-3a was calculated as follows:

Lg (Mw) = −0.2906Rt + 13.124
(1)

where Rt is the retention time of SMP-3a on HPGPC, Mw is the molecular weight of SMP-3a.

### 2.6. Reduction of Carboxyl Groups

The reduction of uronic acid was conducted according to a previously reported method with little modifications [16,17]. SMP-3a (60 mg) was dissolved in 30 mL distilled water, and then 750 mg 1-ethyl-3-(3-dimethylaminopropyl) carbodiimide (EDC) was added. The pH value of the reaction mixture was maintained at 4.75 for 3 h with 0.06 M HCl. Then, 2 M sodium borohydride solution was added slowly to reduce the carbonyl group at room temperature, with pH controlled at 7 with 4 M HCl. Finally, the reaction mixture was dialyzed against distilled water. The retentate was lyophilized and named as SMP-3a-R.

### 2.7. Chemical Components and Monosaccharide Composition Analysis

Total carbohydrate content of SMP-3a was determined by phenol-sulfuric acid method with D-glucose as the standard [15]. Soluble protein content of SMP-3a was measured by the Bradford method using bovine serum albumin as the standard [18]. The content of uronic acid was determined by the sulfuric acid-carbazole method [19].

The monosaccharide composition of SMP-3a and SMP-3a-R were identified and quantified by gas chromatography (GC) analysis according to the reported method with little modifications [20,21], respectively. Briefly, SMP-3a and SMP-3a-R were hydrolyzed with 2 mL 2 M trifluoroacetic acid (TFA) at 100 °C for 6 h. The excess acid was removed by evaporation. Then the hydrolysate was reduced with NaBH_4_ for 3 h followed by neutralization with acetic acid. After the excess boric acid was totally removed by co-distillation with methanol, the residue was acetylated with 1:1 pyridine–acetic anhydride for 1 h at 100 °C. The resulting alditol acetates were analyzed through Shimadzu GCMS-QP 2010 system (Shimadzu, Japan) equipped with a Restek RXI-5 SIL MS column (30 m × 0.25 mm × 0.25 μm). The GC–MS temperature program was increased from 120 °C to 250 °C at 3 °C/min and maintained for 5 min. Standard monosaccharides (rhamnose, fucose, arabinose, xylose, mannose, glucose, and galactose) were derivatized and subjected to GC-MS analysis under the same conditions.

### 2.8. Methylation Analysis of SMP-3a-R

A significant proportion of uronic acid in SMP-3a makes methylation experiments difficult [20], so the carboxyl group of SMP-3a should be reduced prior to methylation analysis. Methylation analysis of SMP-3a-R was performed as previously described by Needs & Selvendran (1993) [22]. Briefly, dried sample (5 mg) was dissolved in 1.5 mL of DMSO. Then, 50 mg NaOH was added into the bottle and sonicated until the sample was completely dissolved. Methyl iodide (0.9 mL) was added to the mixture slowly with ice bath cooling. The reaction was stopped with deionized water. Then, the methylated alditols were isolated by adding methylene chloride and water. The organic phase was washed with water three times and dried by rotary evaporation under a vacuum to obtain the permethylated polysaccharide. The methylated polysaccharide was hydrolyzed with 1 mL 2 M (TFA) for 6 h at 100 °C and reduced with NaBH_4_ for 8 h. Finally, the reduced product acetylated with 1 mL acetic anhydride at 100 °C for 2 h, and the partially methylated alditol acetates (PMAA) were analyzed by GC–MS system (Shimadzu GCMS-QP 2010) equipped with a RXI-5 SIL MS column (30 m × 0.25 mm × 0.25 μm). The temperature program increased from 120 °C up to 250 °C at 3 °C/min and held at 250 °C for 5 min.

### 2.9. NMR Analysis

About 50 mg of SMP-3a was dissolved in 0.5 mL of D_2_O in an NMR tube, and then the ^1^H NMR, ^13^C NMR, NOESY, COSY, HSQC, and HMBC spectra were recorded on a Bruker Ascend 500 MHz spectrometer at room temperature (25 °C).

### 2.10. Scanning Electron Microscopy (SEM) Observation

The microstructure of the SMP-3a was carried out using a S3400N scanning electron microscope (Hitachi LTD., Tokyo, Japan). The freeze-dried SMP-3a was fixed on aluminum tape and coated with a gold layer under reduced pressure. The images were collected at an accelerating voltage of 5.0 kV.

### 2.11. Thermal Gravimetric Analysis (TGA) and Differential Scanning Calorimetric (DSC) Analysis

Thermal gravimetric analysis and differential thermal gravity (DTG) of SMP-3a was detected using a thermogravimetric analyzer (TG-209F1, NETZSCH, Selb, Germany). Seven mg SMP-3a was placed in the sample pan and heated from 30 °C to 500 °C at a rate of 10 °C/min under nitrogen atmosphere. The flow rate of nitrogen was 40 mL/min.

The thermal stability of SMP-3a was determined by differential scanning calorimetry (DSC1, Mettler, Greifensee, Switzerland) and heated from 25 to 300 °C at a rate of 10 °C/min under nitrogen atmosphere.

### 2.12. Immunomodulatory Activity of SMP-3a

#### 2.12.1. Cell Culture

The murine macrophage cell line RAW 264.7 was obtained from the cell bank of Chinese Academy of Science (Shanghai, China). Cells were cultured in DMEM high glucose medium supplemented with 10% FBS, 100 U/mL penicillin, and 100 μg/mL streptomycin at 37 °C in a humidified atmosphere incubator (Thermofisher Scientific Inc., Millersburg, PA, USA) with 5% CO_2_.

#### 2.12.2. RAW264.7 Macrophage Proliferation Assay

The proliferation effect of SMP-3a on RAW 264.7 cells was determined by the CCK-8 method. Briefly, the RAW264.7 cells were incubated in 96-well plate at a density of 1 × 10^5^ cells/mL and cultured for 24 h. Then, the cells were treated with different concentrations (12.5, 25, 50, 100, 200, or 400 μg/mL) of SMP-3a and incubated at 37 °C with 5% CO_2_ humidity environment. LPS (1 μg/mL) was added as positive group. After 24 h, 10 μL of CCK-8 solution was added to each well and incubated at 37 °C for 1 h. The absorbance was detected at 450 nm using a microplate reader (Bio-Tek, Winooski, VT, USA). The proliferation rate of SMP-3a on RAW 264.7 cells was calculated according to the following equation:

Proliferation rate (%) = A_s_/A_c_ × 100% (2)

where A_s_ is the absorbance of the sample, A_c_ is the absorbance of the control.

#### 2.12.3. Measurement of Cytokine Production

RAW264.7 macrophage cells (1 × 10^5^ cells/mL) were seeded in 48-well plate and cultured for 24 h. After the treatment with different concentrations (12.5, 25, 50, 100, 200, or 400 μg/mL) of SMP-3a and 1 μg/mL LPS (positive control) for 24 h at 37 °C, the levels of TNF-α, IL-1β and IL-6 were measured by enzyme-linked immunosorbent assay (ELISA) kits according to the manufacturer’s instructions.

### 2.13. Statistical Analysis

All of the data were presented as the means ± standard deviation (SD) obtained from triplicate experiments. The differences between means were assessed by analysis of variance (ANOVA) with Duncan’s test using SPSS 23.0 software.

## 3. Results and Discussion

### 3.1. Purification of SMP

**Figure 1:**
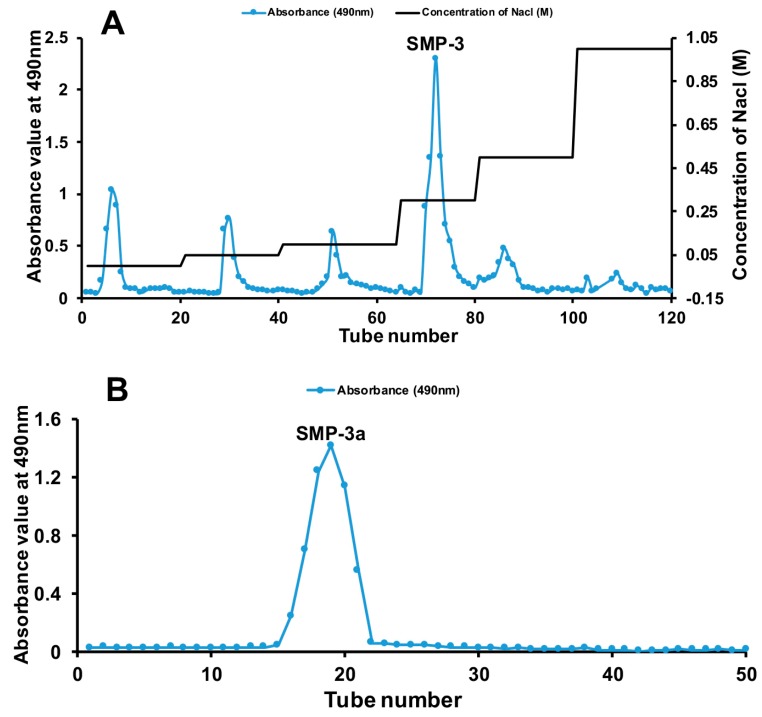
(**A**) Elution profile of SMP on DEAE-52 anion-exchange chromatography with gradient of NaCl solution. (**B**) Elution curve of SMP-3 on Sephadex G-200 gel filtration column with ultrapure water.

SMP was purified by DEAE-52 anion-exchange chromatography with various concentrations of NaCl solutions. One main fraction, SMP-3, was collected through DEAE-52 anion-exchange chromatography (Figure 1A) by elution with 0.3 M of NaCl. As shown in Figure 1B, SMP-3a was finally obtained from SMP-3 in the elution process of the Sephadex G-200 gel filtration column and was chosen for the following study.

### 3.2. FT-IR Spectroscopy Analysis

**Figure 2:**
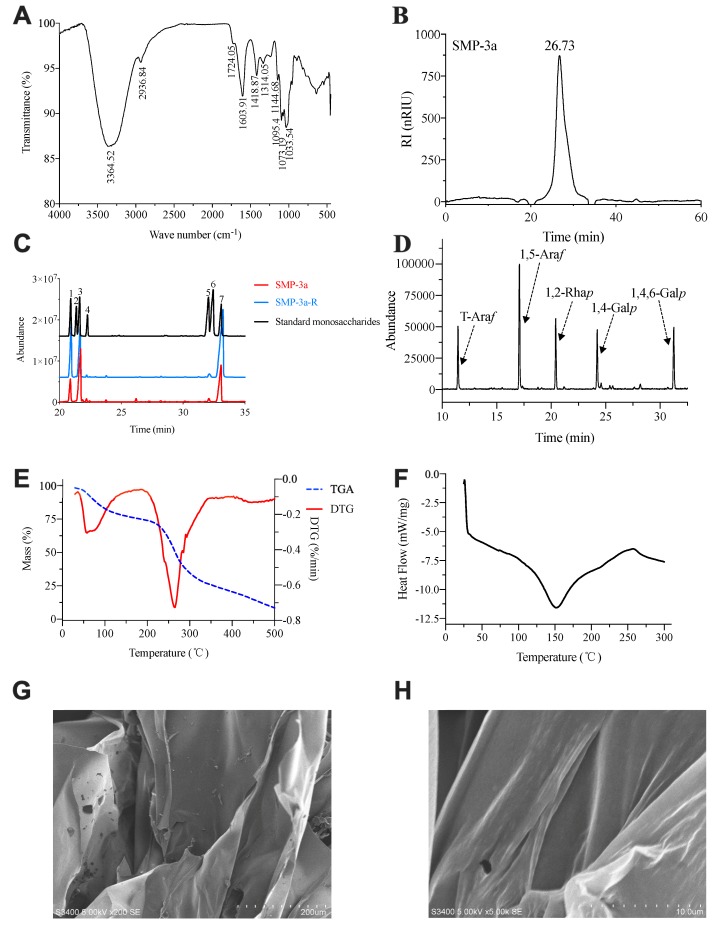
(**A**) The FT-IR spectrum of SMP-3a. (**B**) High performance gel permeation chromatography (HPGPC) of SMP-3a. (**C**) The GC chromatogram profile of SMP-3a, SMP-3a-R and standard monosaccharides. Peak identity: 1. rhamnose, 2. fucose, 3. arabinose, 4. xylose, 5. mannose, 6. glucose, 7. galactose. (**D**) Total ion chromatogram of the methylated product of SMP-3a-R. (**E**) TGA and DTG curve of SMP-3a. (**F**) DSC curve of SMP-3a. (**G**) Scanning electron microscopy images of SMP-3a (200×). (**H**) Scanning electron microscopy images of SMP-3a (5000×).

The FT-IR spectra of SMP-3a ranged from 4000 cm^−1^ to 400 cm^−1^ was displayed in Figure 2A, which showed typical absorption peaks of polysaccharide. The absorption peak at 3417.26 cm^−1^ was assigned to the stretching vibration of O-H. The absorption peak at 2936.84 cm^−1^ was assigned to the C-H asymmetric stretching vibration. The absorption peak 1603.91 cm^−1^ was the bending vibration of O-H [23]. The absorption peak at 1724.05 cm^−1^ was assigned to the carboxyl group, which indicated the presence of uronic acid [24]. The band at 1418.87 cm^−1^ could be due to deforming vibrations of the C-H bond [25]. The absorption peak at 1144.68 cm^−1^ and 1095.4 cm^−1^ were assigned to the α-linkages of arabinofuranoses [26]. The three absorption peaks at 1095.4 cm^−1^, 1073.19 cm^−1^, and 1033.54 cm^−1^ indicated the presence of pyranose ring [27]. Based on the analysis above, it can be concluded that SMP-3a is an acidic polysaccharide containing both pyranose and furanose sugars.

### 3.3. Molecular Weight of SMP-3a

The homogeneity and average molecular weight of SMP-3a was determined by HPGPC. As shown in Figure 2B, the profile of SMP-3a was a single and symmetrically sharp peak, indicating that the SMP-3a was a homogeneous and pure polysaccharide. The average molecular weight of SMP-3a (Mw) was 2.27 × 10^5^ Da (227 kDa), which was calculated according to the calibration curve of standard dextrans.

### 3.4. Chemical Components and Monosaccharide Composition

The contents of carbohydrate, protein, and uronic acid in SMP-3a were found to be 87.62 ± 2.53%, 1.69 ± 0.68%, and 15.18 ± 1.05%, respectively. The identity of uronic acid of SMP-3a was confirmed by comparing the monosaccharide composition between SMP-3a and SMP-3a-R, as shown in Figure 2C. According to the results, SMP-3a was mainly consisted of rhamnose, arabinose, galactose, and galacturonic acid with the molar ratio of 1.09:2.64:1.54:1.

### 3.5. Methylation Analysis of SMP-3a

The carboxyl group of SMP-3a was reduced prior to glycosyl linkage analysis due to the presence of galacturonic acid. The results of methylation analysis were presented in Figure 2D and Table 1. Total ion chromatogram of the methylated products of reduced SMP-3a showed that there were five obvious peaks identified as 2,3,5-Me_3_-Ara*f*, 2,3-Me_2_-Ara*f*, 3,4-Me_2_-Rha*p*, 2,3,6-Me_3_-Gal*p*, and 2,3-Me_2_-Gal*p*, respectively. This indicated that SMP-3a-R was mainly composed of T-linked-Ara*f*, 1,5-linked-Ara*f*, 1,2-linked-Rha*p*, 1,4-linked-Gal*p*, and 1,4,6-linked-Gal*p*. Notably, 1,4-linked-Gal*p* is the reduced form of 1,4-linked-Gal*p*A. The results of methylation analysis revealed that SMP-3a was consisted of Ara*f*-(1→, →5)-Ara*f*-(1→, →2)-Rha*p*-1→, →4)-Gal*p*A-(1→ and →4,6)-Gal*p*-(1→ with the molar ratios of 15.7:32.7:18.2:17.7:15.7.

### 3.6. NMR Analysis of SMP-3a

**Figure 3:**
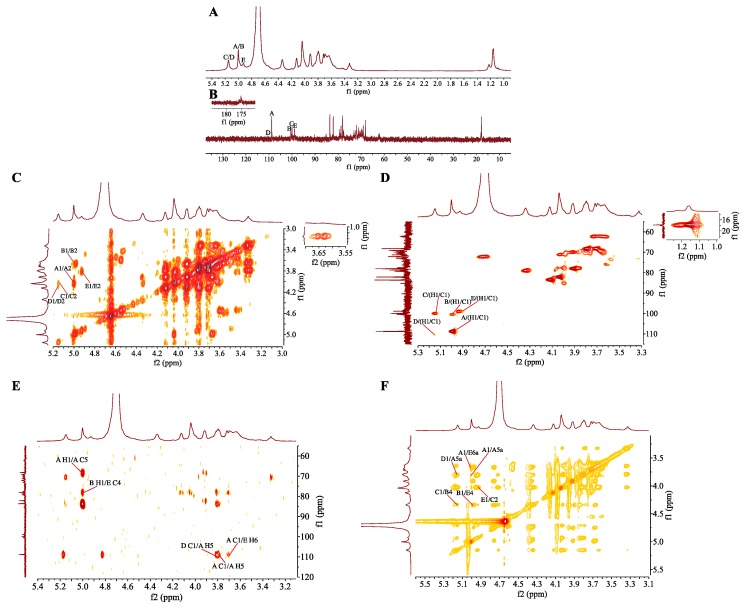
NMR spectrum of SMP-3a recorded at 25 °C (**A**) ^1^H NMR spectrum (500.13 MHz); (**B**) ^13^C NMR spectrum (126 MHz); (**C**) COSY spectrum; (**D**) HSQC spectrum; (**E**) HMBC spectrum; (**F**) NOESY spectrum.

NMR spectroscopy were employed to illustrate the specific structure of SMP-3a. The ^1^H NMR spectrum (Figure 3A) of SMP-3a revealed five anomeric proton signals at δ 5.00, 4.99, 5.15, 5.16, and 4.94 ppm, which were designated as residues A, B, C, D, and E, respectively. As shown in Figure 3B, the corresponding anomeric carbon signals in ^13^C NMR spectrum were δ 108.81, 100.55, 100.07, 110.50, and 98.94 according to their correlation with anomeric proton signals in HSQC (Figure 3D). The strong ^1^H signals at 1.16 ppm and ^13^C signals at 17.83 ppm elucidated the presence of -CH3 group of rhamnose [28]. Signals at 175.13 ppm in the ^13^C NMR corroborated the presence of the uronic acids.

The anomeric proton of residue A was designated at δ 5.00 ppm, which indicated a α-linked residue. In the ^1^H–^1^H COSY spectrum (Figure 3C), the correlative signals at H-1/H-2 (5.00/4.04 ppm), H-2/H-3 (4.04/3.92 ppm), H-3/H-4 (3.92/4.12 ppm), H-4/H-5a (4.12/3.79 ppm), and H-5a/H-5b (3.79/3.72 ppm) gave the chemical shifts of H-2, H-3, H-4, H-5a, and H-5b. The corresponding ^13^C resonances from C-1 to C-5 were determined as δ 108.81, 82.11, 78.05, 83.61, and 68.20 ppm according to their correlation with proton signals in HSQC (Figure 3D). Based on these NMR data and literature [28,29], residue A was deduced as →5-α-l-Ara*f*-1→.

The signal at δ 4.99 ppm was assigned to the anomeric proton signal of residue B, indicating a α-linked residue. The chemical shifts of H-2, H-3, and H-4 and were assigned to δ 3.67, 3.92, and 4.34 ppm based on the cross-peaks H-1/H-2 (4.99/3.67 ppm), H-2/H-3 (3.67/3.92 ppm), and H-3/H-4 (3.92/4.34 ppm) in the COSY spectrum. H-5 was assigned from the cross-peak H-4/H-5 (4.34/4.71 ppm) in the NOESY spectrum. The corresponding chemical shift from C-1 to C-5 were assigned from the HSQC spectrum. The chemical shift of C-6 was assigned to δ 175.13 ppm from the ^13^C NMR spectrum. Residue B was designated as →4)-α-d-Gal*p*A-(1→ [29].

The anomeric proton of residue C was assigned at δ 5.15 ppm, which manifested a α-linked residue. The other protons of residue C were determined from the COSY spectrum and NOESY spectrum. The corresponding carbon signals of C-1 to C-6 were identified on the basis of the correlation between the proton and carbon signals in the HSQC spectrum. In comparison to the reported data [28,29], residue C was deduced as →2)-α-l-Rha*p*-(1→.

As described above, the detailed proton and carbon signals of residue D and E were conducted in the same manner through COSY, NOESY, and HSQC spectra. Based on the previous literatures and data [20,28,29], residue D and residue E were determined as α-l-Ara*f*-1→ and →4,6)-α-d-Gal*p*-(1→, respectively. All the chemical shifts of ^13^C NMR and ^1^H NMR on glycosyl residues (A, B, C, D, and E) were assigned in Table 2 which were derived from COSY (Figure 3C), HSQC, HMBC, and NOESY (Figure 3F) spectra.

HMBC and NOESY spectrum are efficient methods for the analysis of glycosidic linkages between sugar residues [30]. Based on the ^1^H-^13^C HMBC spectrum (Figure 3E), a correlation peak was observed between H-1 (δ 4.99 ppm) of residue B and C-4 (δ 78.4 ppm) of residue E (B H1/E C4), which indicated the presence of →4)-α-d-Gal*p*A-(1→4),6-α-d-Gal*p*-(1→. A cross peak between C-1 of residue D and H-5 of residue A was observed, which suggested the presence of α-l-Ara*f*-(1→5)-α-l-Ara*f*-(1→. Similarly, the correlation peaks at δ 108.81/3.68 ppm (A C1/E H6), δ 108.81/3.79 ppm (A C1/A H5), and δ 5.00/68.20 ppm (A H1/A C5) demonstrated the existence of →5)-α-l-Ara*f*-(1→6), 4-α-d-Gal*p*-(1→ and →5)-α-l-Ara*f*-(1→5)-α-l-Ara*f*-(1→. Then, the cross peaks at δ 4.99/4.35 (B H1/E H4), δ 5.00/3.68 (A H1/E H6a), δ 5.15/4.34 (D H1/A H5a), and δ 5.00/3.79 (A H1/A H5) in the^1^H-^1^H NOESY spectrum proved the results of the HMBC spectrum. In addition, the cross peaks at δ 5.15/4.34 (C H1/B H4) and δ 4.94/4.03 (E H1/C H2) verified the presence of →2)-α-l-Rha*p*-(1→4)-α-d-Gal*p*A-(1→ and →4,6)-α-d-Gal*p*-(1→2)-α-l-Rha*p*-(1→.

Based on the above mentioned analysis, chemical structure of repeating unit of SMP-3a was established. The main chain of SMP-3a was detected to be →2)-α-l-Rha*p*-(1→4)-α-d-Gal*p*A-(1→4)-α-d-Gal*p*-(1→ and the side chain was substituted at *O*-6 of →4,6)-α-d-Gal*p*-(1→. The branch of SMP-3a was composed of α-l-Ara*f*-(1→ and →5)-α-l-Ara*f*-(1→. Furthermore, the specific molecular structure of SMP-3a was deduced as shown below:

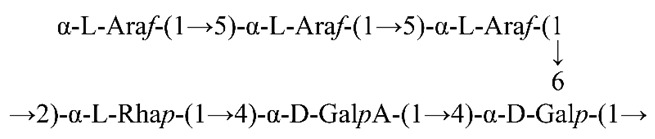


### 3.7. Morphological Analysis

The morphologies of SMP-3a was analyzed by SEM which is a powerful tool to analyze the surface morphology of polysaccharide and the results were displayed in Figure 2. As shown in Figure 2G,H with the different magnification (A: 200×, B: 5000×), the shape of SMP-3a was mainly lamellar or clastic, and the surface was smooth or wrinkle with a compact texture. The flake-like surface of SMP-3a was similar with the polysaccharides from *Cyclocarya paliurus* leaves [31] and purple sweet potato [32].

### 3.8. Thermal Properties

Thermal stability is also an important feature of materials that may have biological applications in view of the potential need for heat sterilization [33]. The thermodynamic properties of SMP-3a was detected by TGA, DTG, and DSC. As shown in Figure 2E, the first mass loss (17.75%) ranged from 31.71 °C to 115.42 °C, for SMP-3a was mainly concerned with the loss of free and bound water, while the second mass loss (45.39%) ranged from 230.44 °C to 367.94 °C was caused by thermal decomposition of SMP-3a. Finally, the T_50_ of SMP-3a was 265.47 °C and the third weight loss was closely related to the oxidation of organic matter. The result indicated that SMP-3a was relatively stable below 230 °C.

The DSC curve (Figure 2F) further illustrated the thermal transition of SMP-3a. An obvious endothermic peak appeared at 150.67 °C, which could be caused by the dehydration or dehydroxylation reactions and the loss of peripheral polysaccharide chains [34]. The above results indicated that the SMP-3a had excellent thermal stability for application in the food and medical industries.

### 3.9. Immunomodulatory Activity of SMP-3a

**Figure 4:**
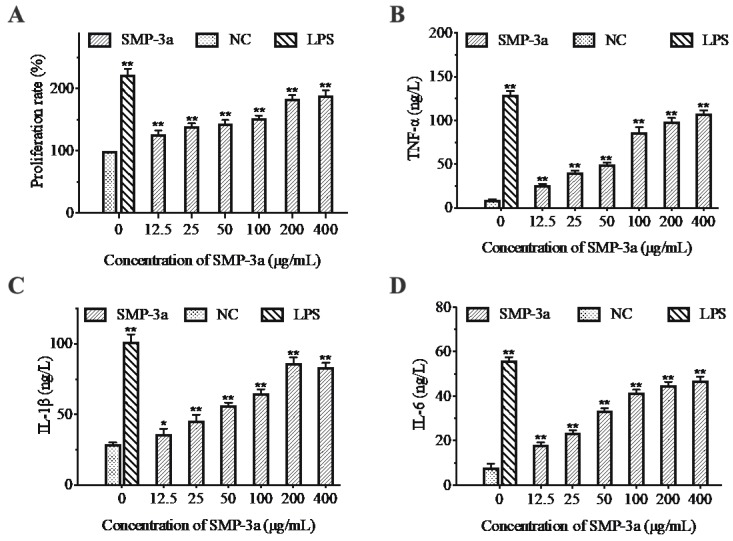
Effect of SMP-3a on the proliferation of RAW264.7 murine macrophage cells (**A**). Effect of SMP-3a on the production of cytokines TNF-α (**B**), IL-1β (**C**), and IL-6 (**D**) in RAW264.7 murine macrophage cells. 1 μg/mL LPS was added as positive group. Data are presented as mean ± SD (n = 3). * *p* < 0.05, ** *p* < 0.01 compared with the negative control (NC).

Macrophages play an important role in the immune system. They can initiate the innate immune response and help fight infection and inflammation [13]. Thus, in the present study, RAW264.7 cells were used to investigate the immunoregulatory activity of SMP-3a in vitro. As shown in Figure 4A, SMP-3a could stimulate RAW264.7 cells proliferation effectively in a dose-dependent manner. Moreover, the cell proliferation rate increased from 126.8 ± 5.6% at 12.5 μg/mL to 189 ± 8.1% at 400 μg/mL.

Cytokines are intercellular signaling proteins with a wide range of biological activities that secreted by both immune and non-immune cells [13]. Macrophages can kill pathogens and regulate immune responses by producing proinflammatory cytokines, such as TNF-α, IL-1β and IL-6. As shown in Figure 4, the production of TNF-α, IL-1β, and IL-6 in the negative control was low. However, the level of TNF-α, IL-1β and IL-6 increased significantly after the treatment with different concentrations of SMP-3a. At the concentration of 12.5–400 μg/mL, the highest production of TNF-α (108 ± 3.6 ng/L) and IL-6 (47 ± 1.7 ng/L) were obtained at 400 μg/mL, while the highest level of IL-1β reached 86.4 ± 4.1 ng/L at 200 μg/mL. The results indicated that SMP-3a could obviously promote the secretion of TNF-α, IL-1β, and IL-6 in RAW 264.7 cells in a concentration-dependent manner.

## 4. Conclusions

A new acid, heteropolysaccharide SMP-3a, was successfully isolated and purified from *Solanum muricatum* pulp. Structural analysis revealed that SMP-3a was mainly consisted of rhamnose, arabinose, galactose, and galacturonic acid with the molar ratio of 1.09:2.64:1.54:1. Methylation and NMR analysis indicated that the backbone of SMP-3a was deduced as →2)-α-l-Rha*p*-(1→4)-α-d-Gal*p*A-(1→4)-α-d-Gal*p*-(1→ with branch at *O*-6 position of →4,6)-α-d-Gal*p*-(1→. The side chain was consisted of α-l-Ara*f*-(1→ and →5)-α-l-Ara*f*-(1→. Preliminary activity tests manifested that SMP-3a exhibited immunomodulatory activity in a dose-dependent manner within the concentration range of 12.5–400 μg/mL. Specifically, SMP-3a enhanced the proliferation of macrophages as well as stimulated macrophages to release proinflammatory cytokines. In summary, SMP-3a could be explored as a potential new source of natural immunomodulator with thermal stability for use in food, healthcare, and medical industries.

## Figures and Tables

**Table 1:** GC–MS analysis of the methylated product (alditol acetate) of carboxyl-reduced SMP-3a.

Retention Time (min)	Methylated Sugar (Alditol Acetate)	Molar Ratios	Type of Linkage	Mass Fragments (*m*/*z*)
11.47	2,3,5-Me_3_-Ara*f*	1	Ara*f*-(1→	71,87,101,117,129,145,161
17.11	2,3-Me_2_-Ara*f*	2.08	→5)-Ara*f*-(1→	71,87,99,101,117,129,161,189
20.45	3,4-Me_2_-Rha*p*	1.16	→2)-Rha*p*-1→	87,101,117,129,143,159,189
24.23	2,3,6-Me_3_-Gal*p*	1.13	→4)-Gal*p*-(1→	87,99,101,113,117,129,131,161,173,233
31.26	2,3-Me_2_-Gal*p*	1	→4,6)-Gal*p*-(1→	71,85,87,99,101,117,127,159,161,201

**Table 2:** Assignments of ^1^H NMR and ^13^C NMR Chemical shift (δ) of SMP-3a on the basis of COSY, HSQC, HMBC, and NOESY spectra (the bold values indicate the linkage position).

Glycosyl Residues	C1	C2	C3	C4	C5	C6	
	H1	H2	H3	H4	H5/H5a	H5b/H6a	H6b
→5)-α-l-Ara*f*-(1→	108.81	82.11	78.05	83.61	68.20		
A	5.00	4.04	3.92	4.12	3.79	3.72	
→4)-α-d-Gal*p*A-(1→	100.55	69.49	69.98	79.04	72.08	175.13	
B	4.99	3.67	3.92	4.34	4.71		
→2)-α-l-Rha*p*-(1→	100.07	77.70	70.70	73.22	70.58	17.83	
C	5.15	4.03	3.78	3.33	3.64	1.16	1.22
α-l-Ara*f*-(1→	110.50	83.40	77.65	79.26	62.21		
D	5.16	4.12	4.03	4.34	3.64	3.72	
→4,6)-α-d-Gal*p*-(1→	98.94	70.80	77.60	78.40	69.12	66.80	
E	4.94	3.81	4.02	4.35	3.83	3.68	3.76
